# Robotic-Assisted Laparoscopic Repair of Petit's Hernia With Preperitoneal Mesh

**DOI:** 10.7759/cureus.63771

**Published:** 2024-07-03

**Authors:** Rubén Neris, Benjamin Yglesias

**Affiliations:** 1 General Surgery, Trumbull Regional Medical Center, Warren, USA; 2 Surgery, Trumbull Regional Medical Center, Warren, USA

**Keywords:** robotic-assisted surgery, mesh placement, petit's hernia, minimal invasive, general surgery

## Abstract

Lumbar hernias are rare abdominal wall hernias that occur in the posterolateral abdominal wall. Intra-peritoneal or extra-peritoneal contents typically protrude through defects in one of two anatomical triangles. The superior lumbar triangle (Grynfeltt-Lesshaft triangle) is an inverted triangle bordered by the 12th rib superiorly, the internal oblique muscle laterally, and the erector spinae muscle medially. The inferior lumbar triangle (Petit's triangle) is an upright triangle bordered by the iliac crest inferiorly, the external oblique muscle laterally, and the latissimus dorsi muscle medially. Surgical repair has been described via open or laparoscopic approach.

A 69-year-old male patient presented with right flank pain and swelling. He was involved in a motorcycle accident 10 months prior, which likely resulted in the development of a traumatic lumbar hernia which was demonstrated on the CT scan. The hernia was clinically incarcerated, and the defect contained the cecum and ileocecal valve. The defect was noted just superior to the iliac crest, by definition, making this an inferior lumbar hernia or a Petit's hernia. The hernia was repaired via robotic-assisted laparoscopic transabdominal approach. A peritoneal flap was created exposing the fascial defect. The fascia was primarily repaired with suture. The defect was reinforced with an 11.4 cm round Ventralight ST mesh in the preperitoneal space.

The patient tolerated the procedure well with no acute complications. He was discharged the same day as an outpatient with appropriate pain control. Short-term follow-up demonstrated no recurrent hernia present and symptoms resolved.

Lumbar hernias are a rare occurrence with no gold standard technique for repair. The benefits of the laparoscopic approach have been described over the open approach. This case report describes utilizing a minimally invasive approach to primarily repair a lumbar hernia defect while also reinforcing the hernia with mesh in the preperitoneal space.

## Introduction

Lumbar hernias are rare abdominal wall hernias that occur in the posterolateral abdominal wall [1]. Intra-peritoneal or extra-peritoneal contents typically protrude through defects in one of two anatomical triangles. The superior lumbar triangle (Grynfeltt-Lesshaft triangle) is an inverted triangle bordered by the 12th rib superiorly, the internal oblique muscle laterally, and the erector spinae muscle medially [2]. The inferior lumbar triangle (Petit's triangle) is an upright triangle bordered by the iliac crest inferiorly, the external oblique muscle laterally, and the latissimus dorsi muscle medially [3]. The floor consists of the aponeurosis of the internal oblique and transversus abdominis muscle. About 80% of lumbar hernias are acquired hernias. These develop either primarily (spontaneous) or secondarily from a trauma or previous operations including nephrectomy, adrenalectomy, abdominal aortic aneurysm repair, iliac bone harvest, etc. [4]. Trauma may lead to the development of lumbar hernias by damaging nerves to the abdominal wall and/or by avulsing musculotendinous structures from their bony attachments, such as the iliac crest [5]. The gold standard for diagnosis is a CT scan, which has a sensitivity of 98%. CT scans can also aid in delineating the defect in fascial and muscular layers and better identify herniated contents [6]. Surgical repair has been described via an open or laparoscopic approach. The advantages of the laparoscopic approach have been demonstrated by Moreno-Egea et al. showing shorter hospital stay, less postoperative pain, and quicker return to activity [7]. A recent study (2020) by Di Giuseppe et al. demonstrated excellent results, with zero cases of conversion to open surgery or recurrence at six-month follow-up after a robotic-assisted laparoscopic approach [8]. In this case report, we describe an incarcerated inferior lumbar hernia secondary to trauma which was repaired via a robotic-assisted laparoscopic approach with primary closure of the defect and placement of preperitoneal mesh.

This report was a poster presentation at the Society of American Gastrointestinal and Endoscopic Surgeons (SAGES) 2021 in Las Vegas, Nevada, United States.

## Case presentation

**Figure 1:**
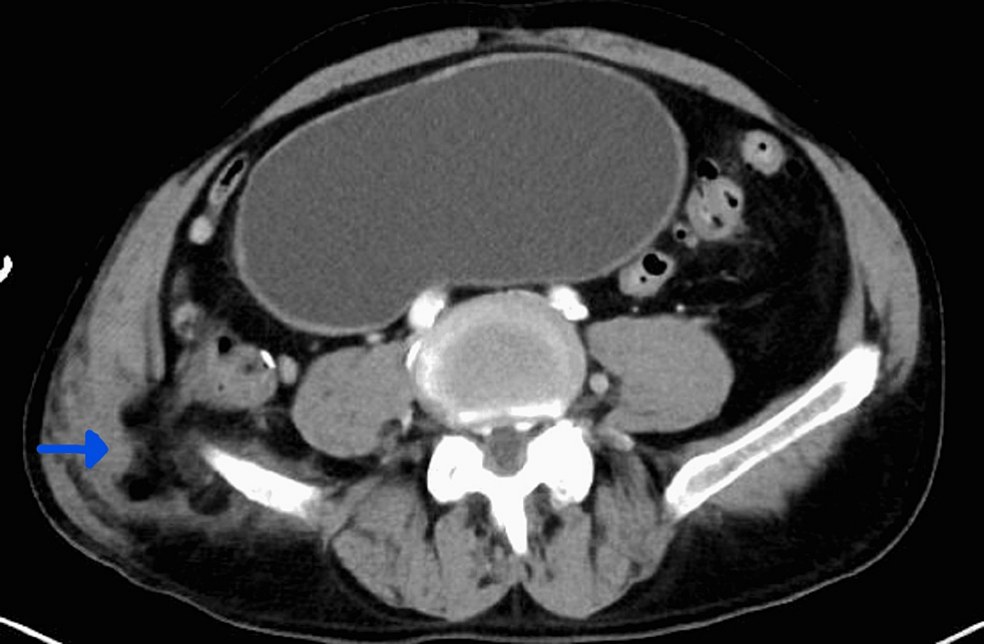
Initial CT of the abdomen/pelvis demonstrating a right lumbar defect containing fat (see blue arrow)

A 69-year-old male patient presented to the general surgery department with complaints of right flank pain and swelling. He was involved in a motorcycle accident 10 months prior, which likely resulted in the development of a traumatic lumbar hernia (Figure 1) demonstrated on a CT scan of the abdomen and pelvis. As seen in Figure 1, the hernia defect only contained fat at this time. The patient was lost to follow-up.

**Figure 2:**
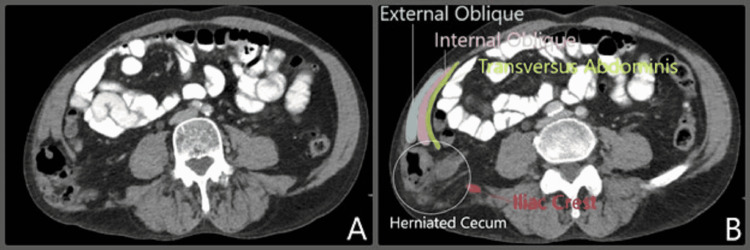
(A) Preoperative CT of the abdomen/pelvis, axial view, demonstrating a right lumbar defect now containing cecum. (B) Same image, structures labeled

**Figure 3:**
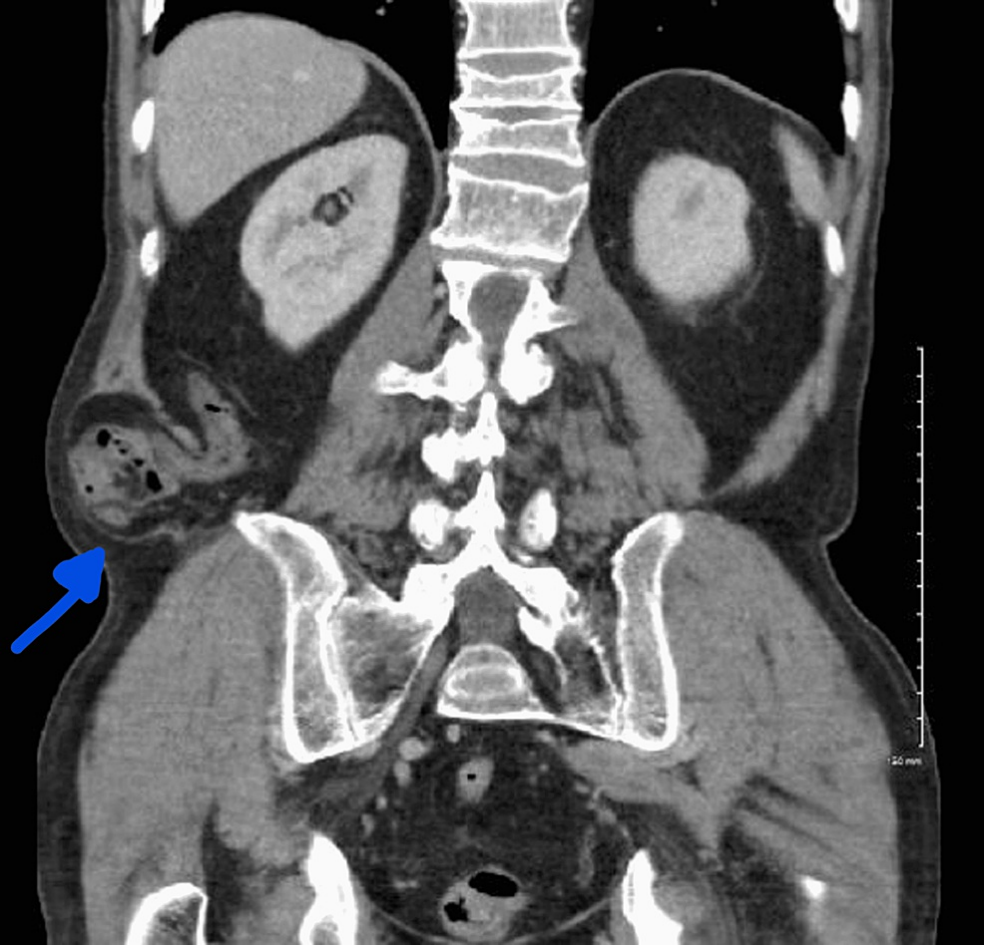
Preoperative CT of the abdomen/pelvis, coronal view, demonstrating a right lumbar defect now containing cecum (see blue arrow)

The patient re-presented with increasing right lower flank pain. The hernia was not reducible, and a preoperative CT of the abdomen/pelvis was obtained (Figure 2 and Figure 3). As seen in Figure 2, the defect now contains the cecum and ileocecal valve and is clinically incarcerated. The defect was noted just superior to the iliac crest, by definition, making this an inferior lumbar hernia or a Petit's hernia. Given these findings, the patient was scheduled for surgical intervention.

**Figure 4:**
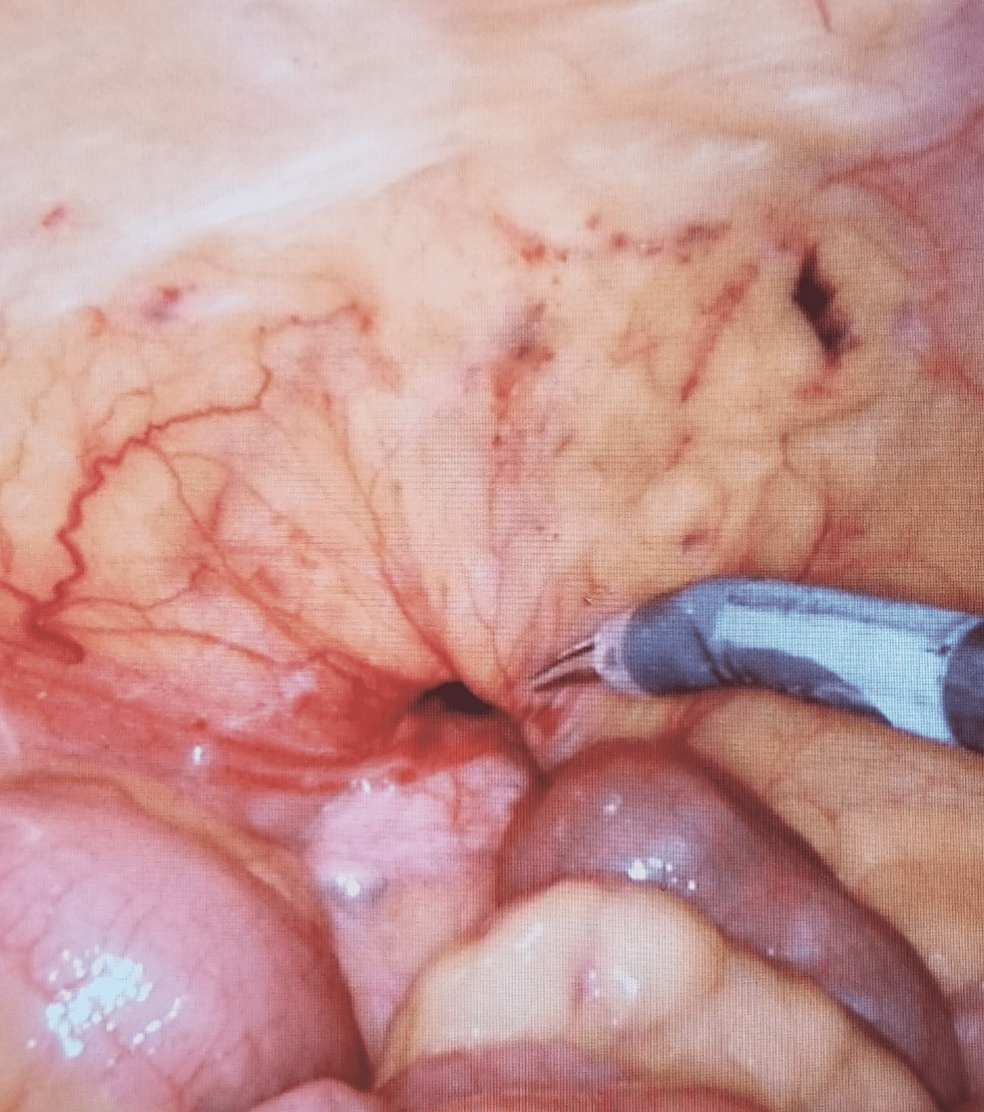
Right inferior lumbar hernia, contents reduced, peritoneum intact

In the operating room, the patient was placed in the supine position with the right hip bumped. An incision was made in the left upper quadrant to permit entry into the abdomen via a 12 mm Optiview port. Once an adequate pneumoperitoneum was achieved, one 8 mm port was placed inferiorly in the left abdomen, while the other was placed in the upper midline. An 8 mm port was then piggy-backed over the 12 mm Optiview port. The patient was then placed in the Trendelenburg position with the right side up to visualize the lumbar hernia containing the cecum and right colon. The contents were easily reduced, and a picture was taken of the fascial defect (Figure 4).

**Figure 5:**
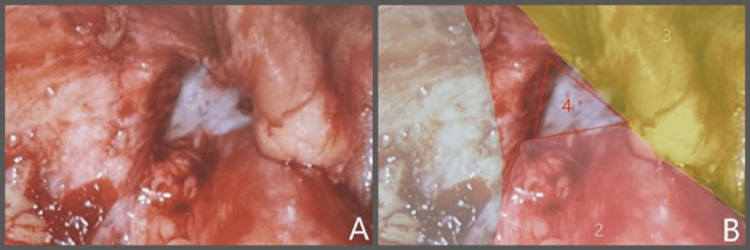
(A) Peritoneum taken down, now exposing fascial defect. (B) Structures labeled: (1) iliac crest, (2) quadratus lumborum, (3) transversalis fascia/transversus abdominis, and (4) upright inferior lumbar triangle defect

**Figure 6:**
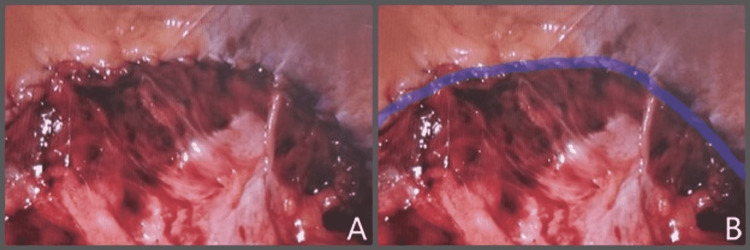
(A) Closure of the peritoneal flap; no mesh is exposed. (B) Same view with the suture line highlighted in blue

Next, the peritoneum was taken down creating a peritoneal flap. The sac was reduced along with its contents. Adequate space was created to allow the placement of the mesh with overlap (Figure 5). The defect measured 6 cm. The fascia was approximated to the iliac bone using an absorbable 0 barbed suture. An 11.4 cm round Ventralight ST mesh with an echo positioning system was inserted and positioned appropriately. The mesh was secured in place with an interrupted absorbable 2-0 vicryl suture. The peritoneal flap was then pulled up completely covering the mesh and approximated using running absorbable 2-0 barbed suture (Figure 6). The pneumoperitoneum was released, and the 12 mm port site was closed using 0 vicryl suture. The patient tolerated the procedure well.

This procedure was completed as an outpatient. The patient was sent home with seven tablets of Percocet and was to follow up in the clinic in one week. At that time, he was back to normal activity. The patient had no recurrence of the hernia or symptoms associated with the hernia.

## Discussion

Lumbar hernias are a rare occurrence, with only 300 cases reported till 2016 [9], and can develop after traumas. Inferior lumbar hernias are most frequently found after blunt trauma [10]. These can result from excessive force to the abdominal wall which can rupture fixation points such as the lateral muscular insertion on the iliac crest. In these cases, the lumbar hernia is present early after the trauma compared to hernias secondary to surgery which develop over time due to muscle denervation and atrophy. When the lumbar hernia is present early, it has been shown that it can be repaired successfully during the initial hospitalization [11]. In our case report, the lumbar hernia was present on the initial trauma CT scans as demonstrated in Figure 1. This hernia could have been repaired at that time, but the surgical team was not consulted until months later.

The goal of surgical repair is to close the defect and reconstruct and/or reinforce the abdominal wall. Factors that make this procedure challenging are defining the fascial edges and the proximity of the bony attachments which can limit your primary repair [12]. Primary closure of the defect can be sufficient in small hernias, but most should be augmented with mesh given the increased tension area and limited strength of the tissues [13]. For mesh, coated mesh for intra-abdominal use or lightweight mesh is recommended. The open and laparoscopic surgical approaches have both been described. A prospective study has demonstrated the advantages of the laparoscopic approach over open [7]. These advantages include better pain control postoperatively, shorter hospital stay, and earlier return to activity. In our case, a laparoscopic approach was taken with robotic assistance. The addition of robotic assistance was made for an easier dissection and better visualization of structures. There was adequate adjacent fascia to the iliac crest to allow for a primary repair of the defect with a slowly absorbable suture. If the fascia is not sufficient to allow for suture fixation, one can use bone anchor fixation [14]. In addition, robotic assistance aided in creating a peritoneal flap to place the mesh in the preperitoneal space (Figure 6) to reduce intra-abdominal adhesions to the mesh. A totally extra-peritoneal approach has also been described for the repair of lumbar hernias to further reduce intra-abdominal injuries and adhesions [15]. Overall, the technique we described gave the patient a durable repair reinforced with mesh and was completed as an outpatient with no acute complications. Further research and long-term follow-up studies are needed to assess the long-term benefits of robotic-assisted lumbar hernia repairs, as well as to contrast transabdominal versus totally extra-peritoneal. 

## Conclusions

Lumbar hernias are a rare occurrence with no gold standard technique for repair. The benefits of the laparoscopic approach have been described over the open approach. This case report describes utilizing a minimally invasive approach to primarily repair a lumbar hernia defect while also reinforcing the hernia with mesh in the preperitoneal space.

Patients can benefit from this approach whenever indications are appropriate. Using this technique of minimally invasive surgery in patients with Petit's hernia, we can decrease the hospital stay length, decrease the amount of pain postoperatively, avoid large open surgical scars, promote faster healing, and shorten the time patients return to activities. We decided to share this case report in order to show the effectiveness and the capacity of the robotic approach in these cases. 

It is true that data collection and investigation are still to be done regarding this innovative surgical approach for the treatment of Petit's hernia, but this case report is an example of its potential. When the resources and the surgical/robotic experience are there, the outcome is outstanding.
